# A computer‐aided diagnosis (CAD) system based on convolutional neural networks for lung cancer diagnosis from 2D [^18^F]‐ PET/CT images

**DOI:** 10.1002/acm2.70285

**Published:** 2025-10-09

**Authors:** Mohammad Karimpour, Neda Taghinezhad, Alireza Mehdizadeh, Mehrosadat Alavi, Tahereh Mahmoudi

**Affiliations:** ^1^ Research Center for Neuromodulation and Pain Shiraz University of Medical Sciences Shiraz Iran

**Keywords:** (18F) fluorodeoxyglucose, computer aided diagnosis (CAD), convolutional neural network (CNN), lung cancer, PET/CT

## Abstract

**Objective:**

This study aims to automatically classify lung conditions into normal, non‐small cell lung cancer (NSCLC), and small cell lung cancer (SCLC) using [^18^F] FDG PET/CT images and deep learning.

**Methods:**

PET/CT scans from 146 patients (1974 scans) were retrospectively analyzed using two strategies: (1) transfer learning with pre‐trained CNNs, and (2) a custom CNN (Res‐SE Net) incorporating residual and squeeze‐and‐excitation (SE) modules. A patient‐based data splitting approach was used to avoid data leakage. Models were trained and validated at the scan level and evaluated at the patient level using majority voting. Grad‐CAM was employed to generate lesion‐localization heatmaps.

**Results:**

Among the seven evaluated CNN models, the proposed Res‐SE Net demonstrated superior performance, achieving an accuracy of 91.67% and a sensitivity of 92.00% in detecting NSCLC, and an accuracy of 90.14% with a sensitivity of 90.00% for distinguishing SCLC cases. When tested on an external dataset, the model attained an accuracy of 98.00% in binary classification (Normal vs. Cancer). In the three‐class classification task, the model achieved an accuracy of 73.02% for NSCLC and 66.26% for SCLC.

**Conclusion:**

These findings demonstrate the potential of Res‐SE Net architecture for accurate multi‐class lung cancer classification using [18F] FDG PET/CT images.

## 1 INTRODUCTION

Lung cancer is the leading cause of cancer‐related deaths worldwide.^1^ It is crucial to have an accurate diagnosis of lung cancer due to its low survival rate.^2^ There are two main types of primary lung cancer: small‐cell lung cancer (SCLC) and non‐small‐cell lung cancer (NSCLC). This category is based on the appearance of cells in malignancy when viewed under a microscope.

There are several established procedures for diagnosing lung cancer, such as pathological assessment and medical imaging. A computed tomography (CT) scan lacks functional information, making it difficult to identify low‐contrast abnormalities.^3^ Positron emission tomography (PET) is commonly used for cancer imaging. The increased metabolic activity of cancer cells leads to greater uptake of radiopharmaceuticals, facilitating functional imaging.^1^ Using ^18^F‐FDG PET/CT for assessing the anatomical and metabolic characteristics of malignancies can increase diagnostic accuracy.^4^ Despite the availability of advanced imaging techniques such as PET‐CT, the current methods for diagnosing tumors may still be prone to errors. One significant drawback is the occurrence of False Positives (FP), which can be caused by inflammation or infection due to other lung diseases. Moreover, PET/CT scans may sometimes result in False‐Negative (FN) findings, particularly in cases of low‐growth lesions (primary or metastatic).^5^ Additionally, the process of investigating vast amounts of data by clinicians could be time‐consuming and monotonous, which can lead to inaccurate diagnosis.

In recent times, Artificial Intelligence (AI) has made remarkable strides in computer aided diagnosis (CAD), particularly in the classification of biomedical images. Various studies have been conducted to improve radiology interpretation time by using computer‐aided detection (CADe) to identify missed tumors,^6^, ^7^, ^8^ detect metastases,^6^ and enhance radiologist sensitivity for recognizing abnormalities.^9^ Presently, tumors are typically diagnosed without the assistance of AI in clinical practice, which leads to issues such as poor reproducibility even among professionals,^10^, ^11^ and the consumption of time and labor. By utilizing computer systems for automatic cancer diagnosis, it can act as a doctor's assistant and reduce errors.^12^

Deep learning has several applications in medical imaging, such as differential pathology diagnosis, pathology detection, and segmentation.^13^ CAD systems can be used in various medical imaging modalities, including nuclear medicine, CT scanning, MRI, and x‐ray radiography.^7^ A deep learning model has been developed to diagnose pneumonia and pneumothorax from chest x‐ray images.^14^ Another system uses residual neural networks to classify lung cancer into its subtypes from CT images.^15^ Additionally, convolutional neural networks have been utilized to identify COVID‐19 in CT scans.^16^ Alves et al.^17^ investigated the use of deep learning in 2D [18F] FDG‐PET/CT to differentiate between benign and malignant lung nodules. Their model achieved a sensitivity of 80.00%, a specificity of 69.23%, and an accuracy of 73.91%. Punithavathy K et al. utilized PET/CT scans to distinguish between NSCLC and healthy patients using several types of conventional machine learning classifiers.^18^ Meanwhile, Han et al.^19^ demonstrated that machine learning techniques combined with radiomic features can also be useful in differentiating between NSCLC subtypes. Unlike radiomics, which has predetermined characteristics, deep learning features are learned in tandem with clinical problems. While previous research has addressed distinguishing healthy individuals from either SCLC or NSCLC cases, developing a model capable of accurately classifying among three categories—normal, SCLC, and NSCLC—remains highly relevant for clinical decision‐making and screening applications.

Therefore, the main objective of this study is to develop and evaluate deep learning models capable of accurately classifying lung images into three clinically significant categories: normal, SCLC, and NSCLC. To achieve this, we implemented and compared two modeling strategies: (1) fine‐tuning several state‐of‐the‐art pre‐trained CNN architectures, and (2) designing a custom CNN model from scratch, incorporating Residual and squeeze‐and‐excitation (SE) blocks for enhanced feature representation. Additionally, we employed class activation mapping techniques to generate heatmaps that visualize model attention and aid in clinical interpretation.

The paper is organized in the following manner: The “Materials and Methods” section explains the data used and the technique employed. The Results section contains the experimental findings. Lastly, the “Discussion and Conclusion” section covers the discussion and conclusion.

## 2 MATERIALS AND METHODS

**FIGURE 1:**
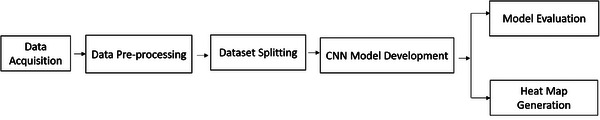
Overall flow diagram of the proposed methodology.

This study consists of five main stages: data gathering, pre‐processing, data splitting, model development, and model evaluation. During the pre‐processing stage, we applied image resizing, normalization, and data augmentation. Data splitting was performed using k‐fold cross‐validation (*k* = 5) to ensure robust performance evaluation. For model development, we fine‐tuned several pre‐trained networks and also designed a custom CNN architecture incorporating Residual and SE attention modules. Model performance was assessed using standard evaluation metrics, and heatmaps were generated to visualize class‐discriminative regions. An overall flow diagram of the proposed methodology is presented in Figure 1.

### 2.1 Dataset

The study included patients who visited Kowsar hospital between June 2019 and March 2025. A total of 146 patients (1974 2‐D scans) were included in the study. Out of these, 933 scans were classified as normal with no signs of malignancy, while 539 scans were diagnosed as NSCLC, and 502 as SCLC. Overview of data collection is presented in Figure S1. To further assess the performance of our model, we evaluated it on cases from The Cancer Imaging Archive (TCIA) that met our inclusion criteria, consisting of 44 patients with 30 NSCLC cases (598 scans) and 14 SCLC cases (378 scans).^20^

### 2.2 PET/CT imaging and labeling

After fasting for 6 h and waiting approximately 60–80 min after receiving an intravenous FDG injection, PET/CT images of the whole body were taken. Although the whole body of the patients was scanned, only the images relevant to the thorax were selected for the study. All image labeling was performed by an experienced specialist in nuclear medicine with over 30 years of clinical experience. For cases that were ambiguous or required further confirmation, the physician referred to the patients’ official medical reports, which were available in their clinical records. These reports, prepared by different attending physicians, provided an additional reference to ensure accurate labeling. In instances of uncertainty, the final labeling decision was made based on a thorough review of both the image data and the corresponding clinical documentation. The PET/CT images of the TCIA dataset were acquired under conditions similar to those used in our study. Patients underwent fasting for at least 6 h, and whole‐body scans were acquired 60 min after intravenous injection of 18F‐FDG. According to the TCIA documentation, the annotation and labeling of images were done by experienced radiologists.^20^

### 2.3 Image preprocessing and augmentation

The Philips scanner provided 2D PET/CT image slices with dimensions of 363 × 657 × 3. To remove unnecessary information, these images were cropped to achieve image sizes of 245 × 457 × 3. The dataset had 1974 2D images, which were divided into a training set (80%) and a test set (20%). The training set was further divided into five folds for cross‐validation. A random sampling process was done at the patient level to prevent biased estimations of test performance. So, there is no data leakage between the training and test set. To enhance the performance of the CNN models and a balance between three groups, the training set underwent data augmentation techniques such as rotation, shift, and zooming. Furthermore, all images underwent normalization using min‐max scaling.

The 2D PET/CT selected images of the TCIA dataset included 1103 images. These images initially had dimensions of 512 × 512 × 3; after cropping to remove unnecessary pixels, the final dimensions became 318 × 512 × 3.

### 2.4 Model development

**FIGURE 2:**
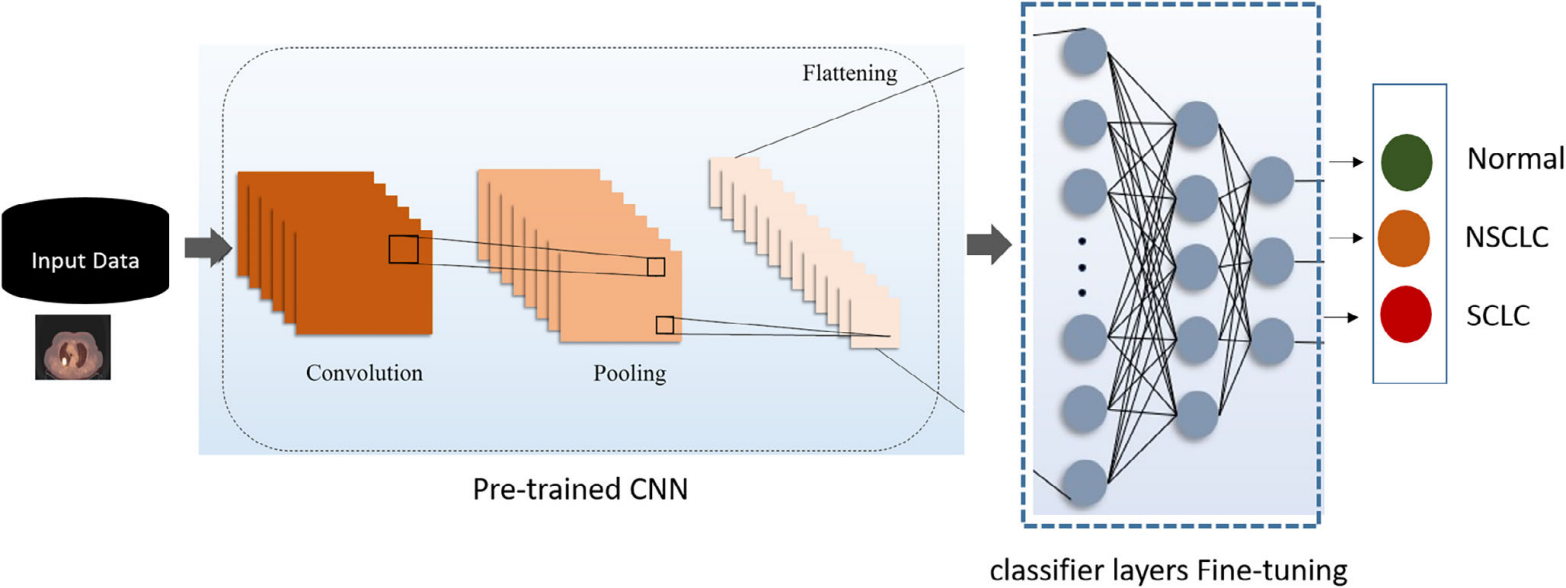
Workflow of the model development using pre‐trained convolutional neural networks. The pre‐trained CNNs include frozen convolutional layers for feature extraction, while the classifier layers are fine‐tuned to perform lung cancer classification.

The Convolutional Neural Network (CNN) is a highly effective algorithm used in computer science.^21^ CNN layers consist of three main types: convolutional, pooling, and fully connected layers. The convolutional layer, a key component of CNNs, identifies input image features by applying kernels. Pooling layers reduce the size of feature maps and network parameters by down‐sampling the output of convolutional layers using pooling kernels. Fully connected layers are responsible for classifying the input image and are typically found at the end of each CNN network design. The output generated by these layers can be used for image categorization (as shown in Figure 2). In this study, we investigated two distinct modeling strategies. The first approach involved the use of well‐established pre‐trained architectures such as ResNet^22^; DenseNet^23^; Inception^24^ and Xception,^25^ while the second focused on the development of a custom‐designed CNN from scratch. The details of both approaches are described in the following sections. The selection of these architectures was driven by their demonstrated efficacy in image classification, architectural diversity, and broad acceptance within the research community.

#### 2.4.1 Model development using pre‐trained CNNs

Transfer learning offers a promising solution to overcome data limitations in machine learning.^26^ It is a technique that involves utilizing the learned features from training a base network on a large dataset and transferring them to a target network. The main idea behind this technique is to apply the knowledge gained from solving a task with abundant labeled data to another task with fewer training examples. By doing this, the target network can benefit from the parameters learned from solving a related task, and its learning process does not start from scratch. In this study, Six CNN models, including InceptionV3, InceptionResNetV2, Xception, DenseNet201, ResNet101V2, and ResNet152V2 were used. These architectures are pre‐trained networks on the ImageNet dataset, which enabled them to learn a diverse set of low‐ and mid‐level features from a large‐scale natural image repository. Specifically, the convolutional base of each model was initialized with ImageNet weights. Then we replaced the last layers of these networks with the designed fully connected networks (FCNs) illustrated in Figure 2. For model adaptation, we customized the degree of fine‐tuning based on the architecture: the initial layers of networks were frozen to preserve the generic features learned on ImageNet, while a subset of higher‐level layers (e.g., the top eight convolutional layers in Xception) were left trainable to adapt to the domain‐specific features of PET/CT images. As shown in Figure 2, the original classification heads were replaced with a custom series of dense layers, including fully connected layers activated by ReLU and a final sigmoid‐activated node for binary/three‐class cancer probability prediction. The input size of the networks was adjusted to 245 × 457 to match the PET/CT image size, while maintaining compatibility with the pre‐trained backbone. Optimization was performed using Adam or SGD optimizers, often with exponential learning rate decay schedules to gradually reduce the learning rate during training. These configurations allowed the model to maintain stability while learning new representations adapted to the PET/CT imaging task. Comprehensive details regarding the fine‐tuning procedures for each network are presented in Table S1.

## 3 MODEL DEVELOPMENT USING CUSTOM CNN (RES‐SE NET)

**FIGURE 3:**
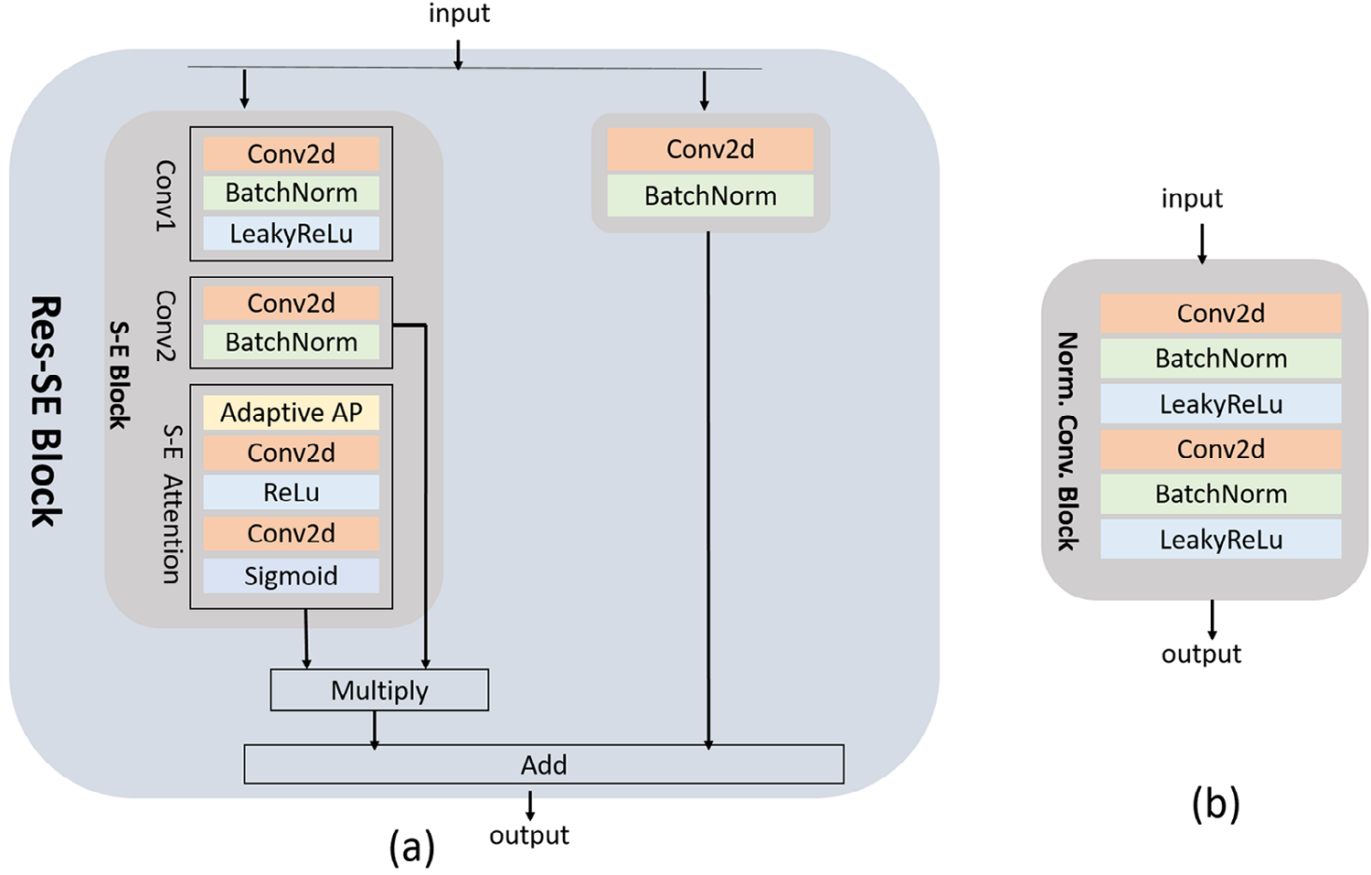
Schematic illustration of two primary building blocks: (a) Res‐SE Block, which integrates residual connections with channel‐wise attention (squeeze‐and‐excitation), (b) Norm. Conv block consists of standard convolutional operations without skip connections.

**FIGURE 4:**
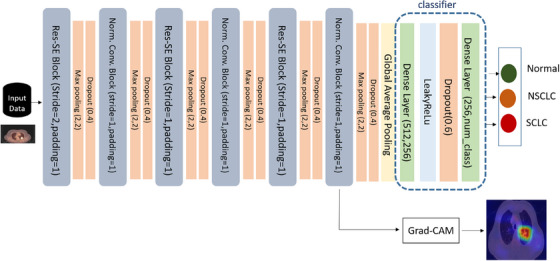
Architecture of the custom‐designed convolutional neural network (CNN) along with heatmap visualization. The model alternates between two types of building blocks: Res‐SE Blocks (Residual blocks with squeeze‐and‐excitation attention) and Normal Convolutional Blocks. These are followed by a global average pooling layer and a fully connected classifier, which maps the extracted features to the final class predictions (Normal, SCLC, NSCLC).

To overcome the limitations observed with standard pre‐trained models in the context of our multiclass lung cancer classification task, we designed and implemented a custom CNN from scratch. The custom‐designed architecture, referred to as Res‐SE Net, integrates two types of convolutional blocks: (1) Residual blocks enhanced with SE attention (Res‐SE Block); Figure 3a, and (2) Standard convolutional blocks without residual connections (Norm. Conv Block); Figure 3b. These two block types are alternated throughout the network to effectively capture both low‐level and high‐level features from the input images. As illustrated in Figure 4, the network begins with a Res‐SE block that reduces spatial dimensions (stride = 2), followed by alternating normal and residual blocks with constant stride. Each block is followed by MaxPooling and Dropout2D layers to improve generalization and reduce overfitting.

### 3.1 Residual‐SE block

Residual connections were introduced to address the degradation problem in deep neural networks. In a residual block, the input is directly added (via a skip connection) to the output of the convolutional layers. This shortcut pathway allows gradients to flow more effectively during training and helps preserve low‐level features across the network layers.^27^ To enhance the discriminative capacity of the network, particularly in highlighting diagnostically relevant image regions, we embedded SE attention modules within each residual block.^28^ The SE mechanism introduces channel‐wise attention by first compressing spatial information using global average pooling (squeeze), and then recalibrating each channel with learned importance weights (excitation). This enables the network to focus more strongly on feature channels that are critical for identifying disease‐relevant patterns. As illustrated in Figure 3a, the original feature maps obtained after two convolutional layers were rescaled using the learned channel‐wise weights from the SE attention block. This mechanism effectively enhances feature channels that are most relevant to the classification task—such as those associated with tumor characteristics—while suppressing less informative or redundant features. This mechanism introduces minimal computational overhead while enabling the model to dynamically focus on the most diagnostically relevant patterns, a critical aspect when dealing with subtle visual differences among cancer types. The details of the SE blocks are presented in Figure 3a.

### 3.2 Standard convolutional block

Each Res‐SE Block in the proposed architecture is followed by a standard Convolutional block (Norm. Conv Block), which consists of two sequential 3×3 convolutional layers, each followed by batch normalization and LeakyReLU activation. Unlike the residual blocks, these standard blocks do not include skip connections or attention mechanisms. This alternation between Res‐SE and Norm. Conv blocks contributes to a richer feature representation, enabling the network to better distinguish between small cell, non‐small cell, and normal lung tissue patterns.

Following the final convolutional stage, a global average pooling layer compresses the spatial dimensions to 1 × 1, producing a 512‐dimensional feature vector (see Figure 4). This vector is then passed through a fully connected classification head comprising a dense layer (512→256) with LeakyReLU activation and dropout (*p* = 0.6), followed by a final dense layer (256→num of classes) that outputs softmax probabilities corresponding to the three classes: normal, SCLC, and NSCLC. The custom CNN model was trained for 200 epochs using a batch size of 64, a learning rate of 0.001with a weight decay of 1e‐4 for regularization.

### 3.3 Heat map generation using Grad‐CAM

To visualize class‐specific attention and identify diagnostically relevant regions in the input images, we applied Gradient‐weighted Class Activation Mapping (Grad‐CAM).^29^ This method analyzes the sensitivity of the model's predictions to various spatial regions within the image by computing gradients with respect to the feature maps of the final convolutional layer. Grad‐CAM generates a heatmap that highlights the image regions most influential to the model's decision—typically corresponding to abnormal tissue structures or lesions. These visual explanations not only help clinicians interpret the AI's focus but also validate that the model is attending to anatomically and pathologically meaningful areas. Importantly, the effectiveness of Grad‐CAM depends on the spatial resolution of the final convolutional feature maps. If excessive pooling or downsampling occurs in the network, the resulting maps may lose critical localization detail. Therefore, in our model, we carefully preserved spatial information by maintaining a sufficiently high‐resolution feature map in the last convolutional layer before global average pooling. This design ensures that the generated heatmaps retain meaningful spatial correspondence to the original image—enabling reliable visualization of potential disease areas. Technically, we computed the gradients of the predicted class score with respect to the selected convolutional feature maps. These gradients were then globally averaged across spatial dimensions to obtain importance weights for each channel. By performing a weighted sum of the feature maps using these weights, we obtained a class‐specific heatmap. The final heatmap was then upsampled to match the resolution of the input image. Notably, this process can also be effectively implemented using the pytorch_grad_cam library.

### 3.4 Patient‐level classification

**FIGURE 5:**
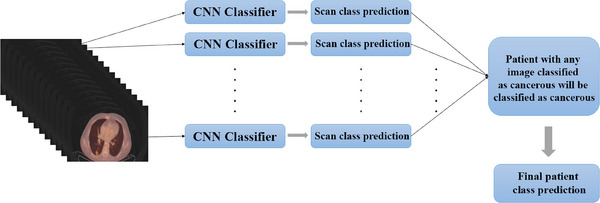
Patient‐level prediction using majority voting. The final label for each patient is determined based on the most frequent prediction across all corresponding scan slices.

In this study, the proposed networks were primarily trained and evaluated on individual scan‐level (slice‐based) images. However, for clinical relevance and robust performance assessment, we additionally performed a patient‐based evaluation. In this approach, for each patient, the model generates predictions for all corresponding image slices. A majority voting strategy is then applied to determine the final predicted label for the patient. An illustration of this patient‐level decision‐making process is provided in Figure 5.

## 4 RESULTS

We implemented the pre‐trained CNN models using the Keras framework with TensorFlow as the backend, while the custom Res‐SE Net architecture was developed and trained using the PyTorch library. To assess the effectiveness of the proposed classification methodology, we used various metrics such as accuracy, sensitivity, specificity, and area under the receiver operating characteristic (ROC) curve (AUC). For all the model performance results the thresholding approach for sensitivity and other metrics is argmax‐based thresholding, where the predicted class is the one with the highest predicted probability. This means a fixed implicit threshold derived from the maximum probability is used rather than an explicit probability threshold for each class. Furthermore, we calculated AUC for each class using one‐vs‐rest ROC curves leveraging the predicted class probabilities.

**FIGURE 6:**
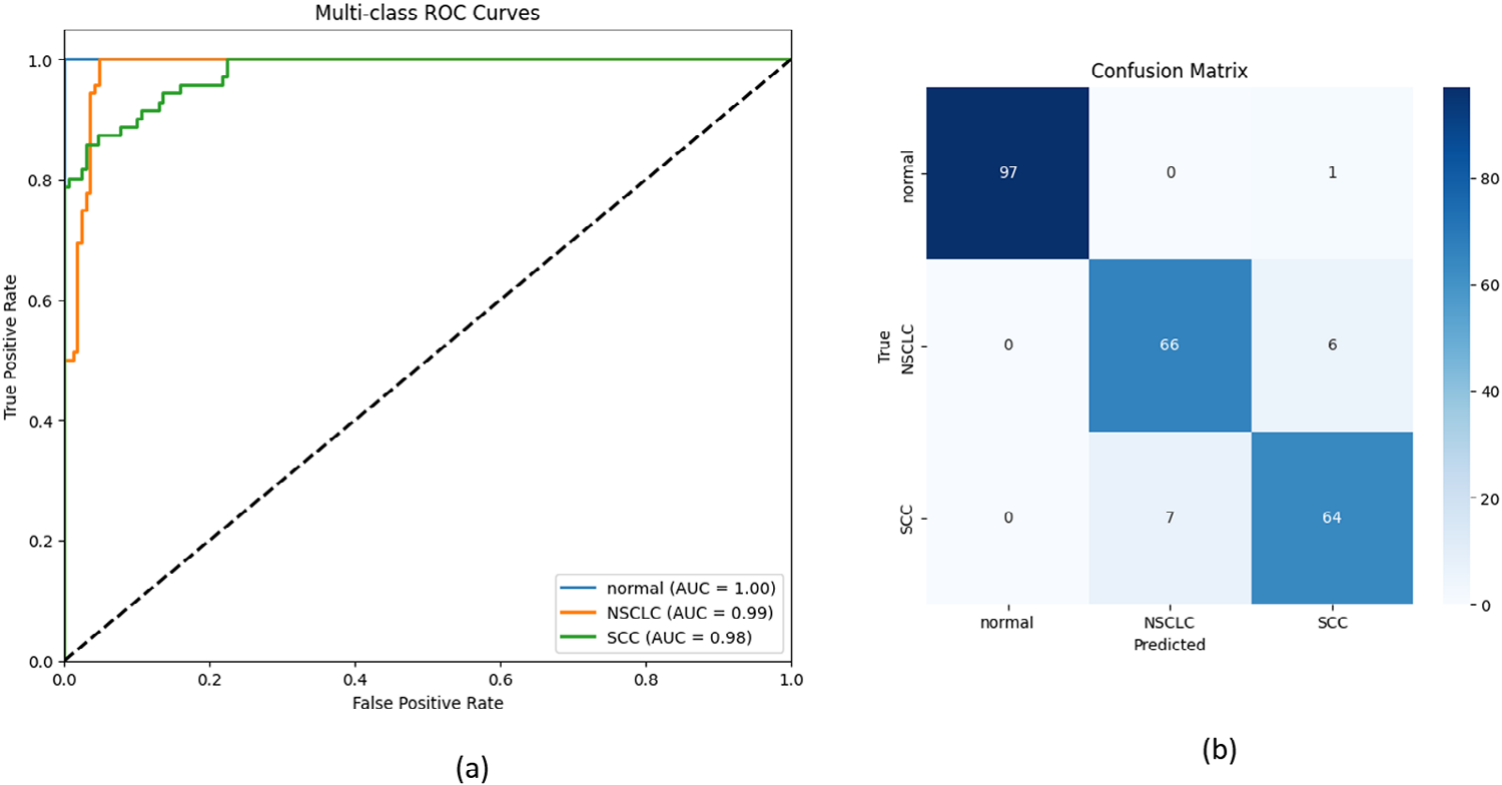
Performance of the proposed Custom Res‐SE Net model. The ROC curve (a) illustrates the model's ability to distinguish between classes, while the confusion matrix (b) shows the classification results on the test set, highlighting the distribution of true and predicted labels.

**FIGURE 7:**
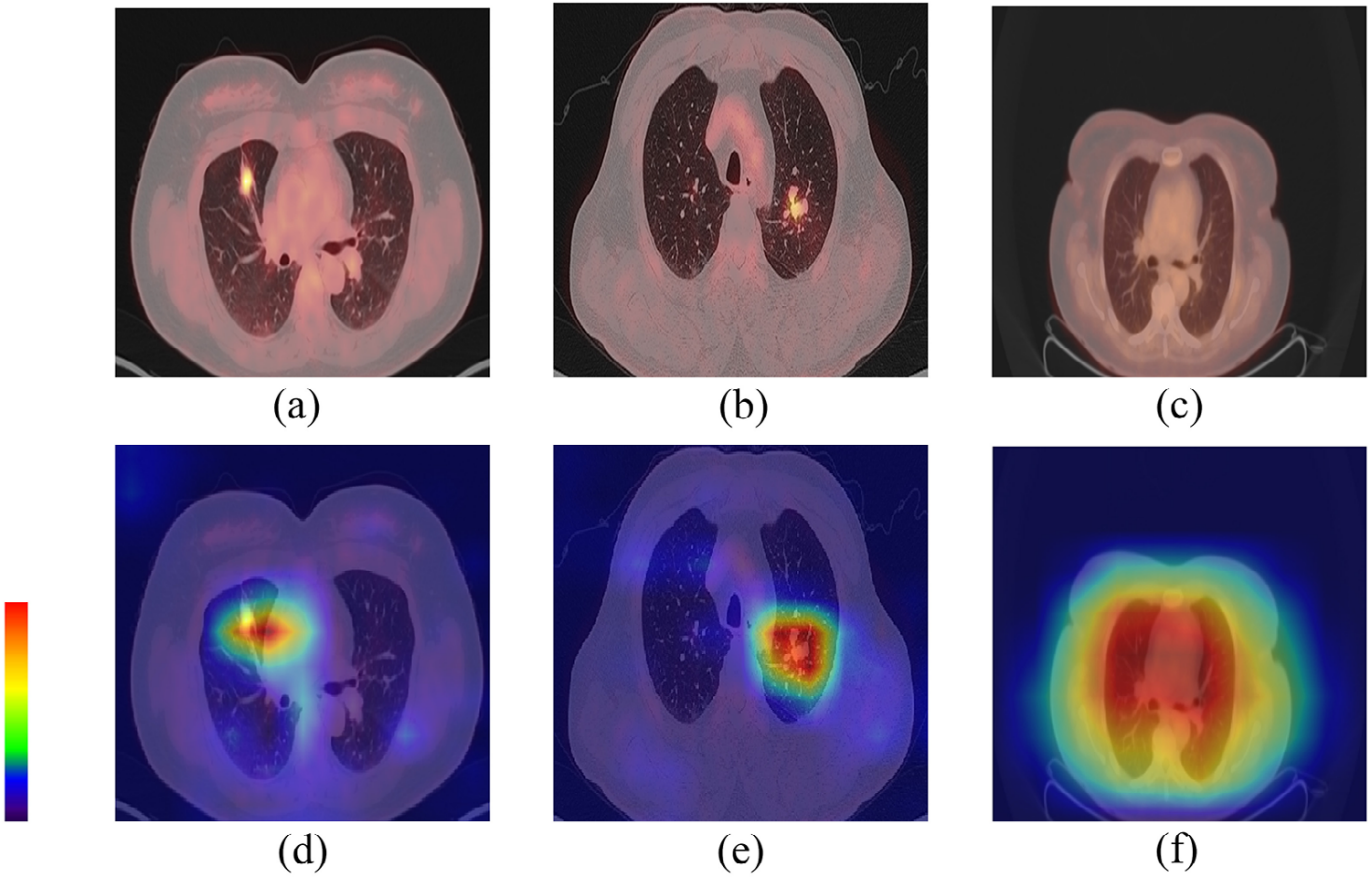
Class‐specific regions of the input images visualized using heatmaps with color intensities ranging from dark blue to red. Red areas indicate regions with strong relevance to the predicted class. (a, d) Sample from an NSCLC case and its corresponding heatmap; (b, e) Sample from an SCLC case and its corresponding heatmap; (c, f) Sample from a normal case and its corresponding heatmap.

In addition to the primary three‐class classification task, we also conducted a binary classification (cancer vs. healthy) as an initial baseline. The experimental results using pre‐trained and our custom Res‐SE models for three‐class classification are reported in Table 1. The full results of the binary task using pre‐trained and our custom Res‐SE models, are also provided in Table 2 and the Figure S2. An effective way to assess the performance of diagnostic tests is by plotting the true positive rate (TPR) against the false positive rate (FPR) on a graph, which is called the ROC curve. The diagnostic accuracy of a study is enhanced when the AUC is closer to 1.^30^ So, we obtained the ROC curve and confusion matrix, as indicated in Figure 6. For further evaluation of the results, heat maps were generated for normal, NSCLC and SCLC cases using Grad‐CAM approach (Figure 7).

**TABLE 1:** Performance of CNN models for three‐class lung cancer classification (Normal, NSCLC, SCLC). The table summarizes the evaluation metrics obtained for each model on the test set, highlighting their ability to differentiate between healthy cases and two major lung cancer subtypes.

**Model**	**Accuracy**	**Precision**	**Sensitivity**	**Specificity**	**AUC**
Xception model	88.91	85.84	93.71	83.91	93.23
ResNet101V2 model	88.17	87.32	89.85	86.43	93.64
ResNet152V2 model	89.90	88.78	91.78	87.93	95.90
InceptionResNetV2 model	81.77	81.22	83.57	79.89	89.68
InceptionV3 model	91.13	89.76	93.23	88.94	95.20
DenseNet201 model	83.49	81.25	87.92	78.89	90.18
**Our proposed Res‐SE Net**	**100**	**100**	**100**	**100**	**1**
**Our proposed Res‐SE Net (External Test)**	**98.00**	**99.89**	**98.36**	**98.97**	**99.52**

**TABLE 2:** Performance of CNN models for two‐class lung cancer classification (Normal and lung cancer (NSCLC/SCLC)). The table summarizes the evaluation metrics obtained for each model on the test set, highlighting their ability to differentiate between healthy cases and two major lung cancer subtypes.

	NSCLC				SCLC				Normal			
Network	Accuracy	Sensitivity	Specificity	AUC	Accuracy	Sensitivity	Specificity	AUC	Accuracy	Sensitivity	Specificity	AUC
InceptionV3	78.36	67.35	83.57	0.88	73.44	66.06	77.55	0.82	94.43	86.73	98.07	0.99
InceptionResNetV2	76.39	50.00	88.89	0.88	74.43	74.31	74.49	0.85	97.38	97.96	97.10	0.99
Xception	74.10	45.92	87.44	0.81	72.79	67.89	75.51	0.75	94.10	97.96	92.27	1
DenseNet201	75.08	47.96	87.92	0.80	73.77	78.90	70.92	0.83	97.38	91.84	100.00	0.99
ResNet152V2	71.80	19.39	96.62	0.85	68.85	92.66	55.61	0.86	96.39	90.82	99.03	1
**Our proposed Res‐SE Net**	**91.67**	**92.00**	**95.85**	**0.99**	**90.14**	**90.00**	**95.88**	**0.98**	**98.98**	**99.00**	**100**	**1**
**Our proposed Res‐SE Net (External Test)**	**73.02**	**73.08**	**54.35**	**0.73**	**66.26**	**41.99**	**78.88**	**0.69**	**98.49**	**98.98**	**98.44**	**1**

We evaluated the patient‐level performance using our proposed Res‐SE Net, which achieved the highest classification accuracy in the previous experiments. For each patient in the test dataset, predictions were generated for all corresponding scans, and the final label was determined through majority voting. This approach resulted in 100% accuracy, sensitivity, and specificity at the patient level. Although a few individual scans were misclassified, the aggregated decision consistently matched the ground truth, confirming the robustness and clinical reliability of the Res‐SE Net model.

As previously mentioned, several images from the TCIA dataset were used to assess the models’ performance on an unseen external dataset. Since all these images were from patients with lung cancer, and no normal images were included, metrics such as precision, specificity, and area under the ROC curve (AUC) could not be calculated. Models were tested on this external dataset, and only those demonstrating significant performance are reported here. The InceptionV3, ResNet152V2, and Xception and our proposed model for distinguishing lung cancer achieved classification accuracies of 80.22%, 77.43%, and 76.83%, and 98.00%, respectively. Furthermore, the model reached an accuracy of 73.2% for NSCLC, while the performance for SCLC was notably lower, reflecting the greater clinical challenge in detecting this subtype.

## 5 DISCUSSION AND CONCLUSION

Accurate diagnosis of lung cancer is crucial for providing appropriate care and treatment to patients. Medical imaging tests are commonly used for cancer diagnosis, and PET/CT imaging is a popular method that provides both anatomical and functional information. However, cancer diagnosis from medical images has limitations and is susceptible to errors. Fortunately, recent developments in AI, particularly in medicine, have shown promising results. Accordingly, a computer‐aided diagnosis system has been designed to diagnose lung cancer from 2D PET/CT images.

In this study, we developed and evaluated deep learning models for the three‐class classification of lung cancer using [18F] FDG‐PET/CT images, aiming to differentiate between normal, NSCLC, and SCLC. Additionally, we developed a binary classification model to distinguish healthy individuals from patients with lung cancer.

In the model development phase, pre‐trained networks performed well in binary classification tasks (normal vs. cancer); however, they failed to deliver acceptable performance in the more challenging three‐class classification, particularly in distinguishing between NSCLC and SCLC. This limitation can be attributed to two main factors: (1) the dataset for NSCLC and SCLC was relatively small, limiting the network's ability to learn class‐specific features; and (2) pre‐trained networks, while powerful, are often overly complex for small, imbalanced datasets, which can lead to overfitting or poor generalization.

To address this, we required a model that is not only lightweight but also capable of focusing on lesion‐relevant features. Therefore, we designed a custom CNN architecture combining residual connections and SE attention modules. The residual connections help reduce optimization difficulties in deeper networks, while the SE blocks enhance the network's ability to focus on the most informative feature channels. This hybrid architecture—Res‐SE Net—proved more effective in distinguishing between normal, NSCLC, and SCLC classes in our dataset. The purpose of inserting Norm. Conv Block between Res‐SE Blocks is twofold: (1) They allow the network to further refine the feature maps extracted by the preceding residual‐attentive block, adding nonlinear transformations without increasing architectural complexity. (2) They help to balance model regularity and depth, preventing over‐dependence on shortcut connections, and enabling the network to learn both enhanced (attention‐weighted) and plain hierarchical features. This design enables the network to retain spatial and contextual information more effectively, enhance feature representation by emphasizing informative channels, and maintain stable gradient flow in deeper layers. Compared to standard pre‐trained models, Res‐SE Net demonstrated competitive performance, particularly in the multiclass setting, while offering a more interpretable and architecture‐tailored solution for PET/CT‐based lung cancer classification.

A notable strength of the proposed CAD system lies in its ability to analyze PET/CT images at both the slice and patient levels. This dual‐level analysis reduces the likelihood of overlooking small lesions, which often contribute to false‐negative results in standard radiological assessments. Furthermore, by generation of heatmaps, the system localizes cancerous regions, providing interpretable visualizations that can assist clinicians in verifying model outputs.

To further examine the generalizability of the Res‐SE Net, we evaluated the model using NSCLC and SCLC cases from the TCIA dataset, combined with normal cases from our internal cohort. The model achieved high accuracy for normal detection (98.49%, AUC = 1.0), confirming its robustness in discriminating cancer versus non‐cancer cases. For NSCLC, the accuracy was 73.02% (AUC = 0.73), whereas performance for SCLC was lower (accuracy 66.26%, AUC = 0.69). These findings suggest that while the proposed model generalizes well for binary cancer detection, distinguishing between NSCLC and SCLC across different cohorts remains challenging. The relatively limited number of external SCLC cases, together with overlapping imaging features of SCLC and NSCLC, may have contributed to this reduced performance. Future work with larger and more balanced multi‐center datasets is required to improve subtype classification.

Several recent studies have explored the application of deep learning in classifying lung cancer subtypes; however, most focus either on binary classification^18^ or on distinguishing NSCLC subtypes without including SCLC or healthy controls.^31^, ^32^

Punithavathy K et al.^18^ utilized machine learning techniques to classify NSCLC and healthy populations. As SCLC is known to be the most aggressive type of lung tumor with high metastatic potential, making diagnosis a crucial problem. Besides, machine learning algorithms require handcrafted features, which is a laborious process that is sensitive to changes in image acquisition, pre‐processing, and feature calculation. So, comparison with existing literature affirms the competitive performance of the proposed model. Prior studies utilizing deep learning for lung cancer diagnosis from PET/CT images reported accuracies ranging from 73% to 89%.^17^, ^18^ The higher accuracy achieved in this study can be attributed to the development of our proposed Res‐SE Net, data augmentation, and careful patient‐level data splitting, which mitigated data leakage and overfitting.

Despite these promising results, several limitations must be acknowledged. The dataset size, while comprehensive, remains relatively small, potentially limiting the model's capacity to generalize to rare lung cancer subtypes. Additionally, integrating clinical data, such as patient history and biomarkers, with imaging data could lead to more holistic diagnostic models. In conclusion, this study presents a robust CAD system capable of accurately diagnosing lung cancer from 2D PET/CT images, with the potential to augment clinical workflows and reduce diagnostic errors. Continued advancements in deep learning (3D classification) and access to larger, diverse datasets will be pivotal in translating this technology into routine clinical practice.

## AUTHOR CONTRIBUTIONS

Mohammad Karimpour was involved in software design, validation, and writing the paper. NT involved in algorithm implementation. Tahereh Mahmoudi and Alireza Mehdizadeh was involved in supervision, conceptualization, methodology, and checking the final version of the manuscript. Mehrosadat Alavi participated in conceptualization, data collection, and checking the labels. All authors read the final version of the manuscript and approved it.

## CONFLICT OF INTEREST STATEMENT

The authors declare no conflicts of interest.

## ETHICS APPROVAL

This study was approved by the Shiraz University of Medical Sciences Institutional Review Board (IRB) (IR.SUMS.REC.1401.715) and followed the tenets of the Declaration of Helsinki.

## Supporting information



Supporting Information

Supporting Information

Supporting Information

Supporting Information

## Data Availability

The data that support the findings of this study are available from the corresponding author upon reasonable request. The full implementation details and source codes for this study are publicly available at our GitHub repository: https://github.com/MKarimpour1997/thesProjectPET‐CT
