# Supplementary figures and images for: A computer‐aided diagnosis (CAD) system based on convolutional neural networks for lung cancer diagnosis from 2D [18F]‐ PET/CT images
Source: J Appl Clin Med Phys. 2025 Oct 9;26(10):e70285. doi: 10.1002/acm2.70285 (PMC12509238; doi:10.1002/acm2.70285)

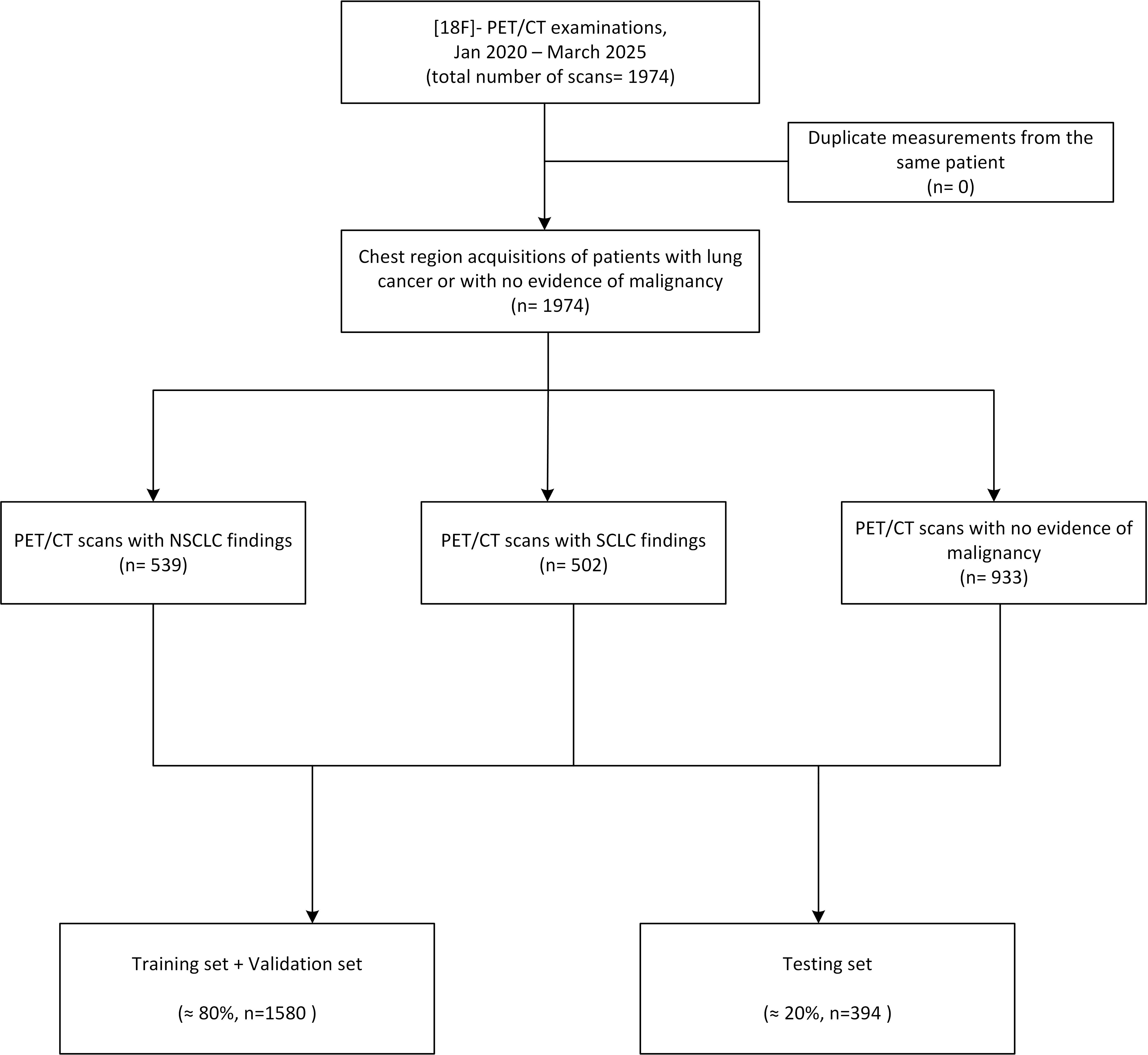

Supplement: Supplementary file 1 — Supporting Information [file ACM2-26-e70285-s004.jpg]

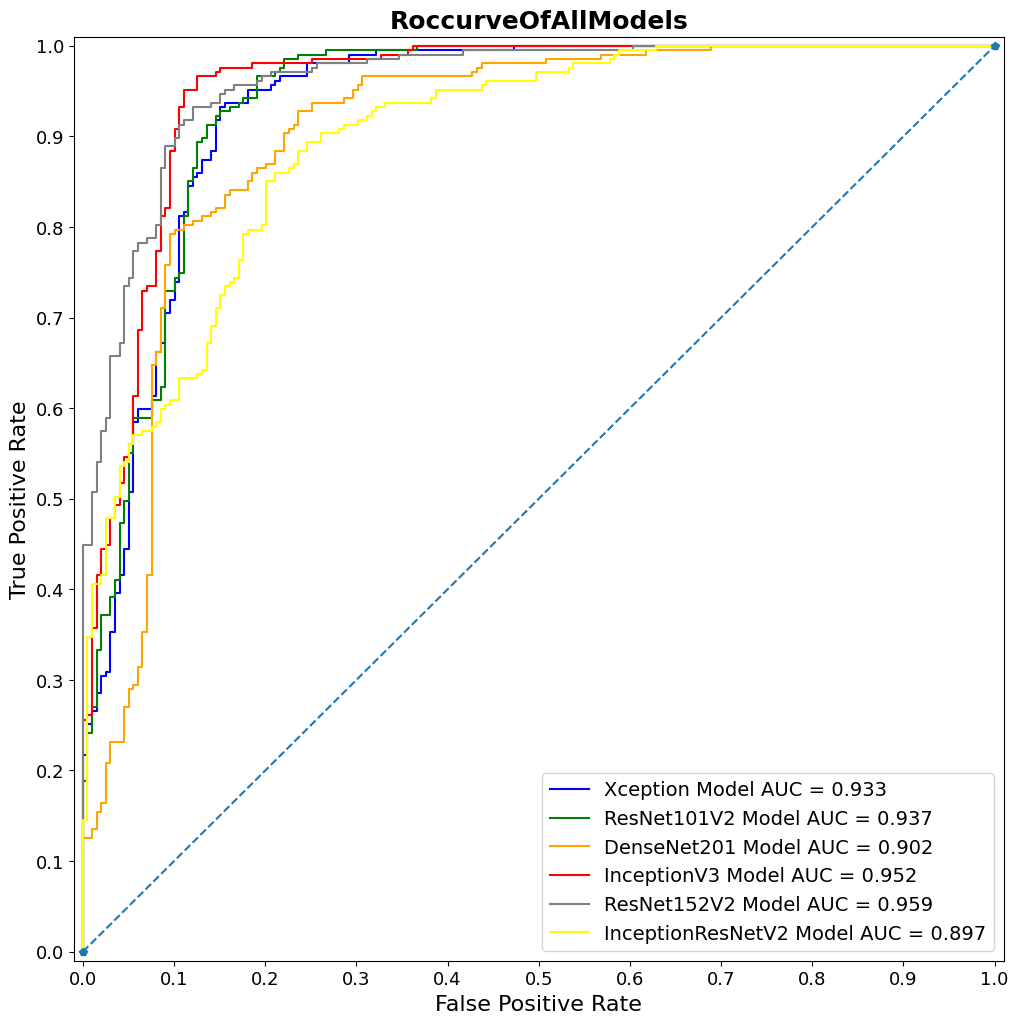

Supplement: Supplementary file 2 — Supporting Information [file ACM2-26-e70285-s003.png]
