# Supplementary material for: A computer‐aided diagnosis (CAD) system based on convolutional neural networks for lung cancer diagnosis from 2D [18F]‐ PET/CT images
Source: J Appl Clin Med Phys. 2025 Oct 9;26(10):e70285. doi: 10.1002/acm2.70285 (PMC12509238; doi:10.1002/acm2.70285)
Supplement: Supplementary file 3 — Supporting Information [file ACM2-26-e70285-s001.docx]

| **Model** | **Accuracy** | **Precision** | **Sensitivity** | **Specificity** | **AUC** |
| --- | --- | --- | --- | --- | --- |
| Xception model | 88.91 | 85.84 | 93.71 | 83.91 | 93.23 |
| ResNet101V2 model | 88.17 | 87.32 | 89.85 | 86.43 | 93.64 |
| ResNet152V2 model | 89.90 | 88.78 | 91.78 | 87.93 | 95.90 |
| InceptionResNetV2 model | 81.77 | 81.22 | 83.57 | 79.89 | 89.68 |
| InceptionV3 model | 91.13 | 89.76 | 93.23 | 88.94 | 95.20 |
| DenseNet201 model | 83.49 | 81.25 | 87.92 | 78.89 | 90.18 |
| **Our proposed Res-SE Net** | **100** | **100** | **100** | **100** | **1** |
| **Our proposed Res-SE Net (External Test)** | **98.00** | **99.89** | **98.36** | **98.97** | **99.52** |

Table 2. Performance of CNN models for two-class lung cancer classification (Normal, lung cancer (NSCLC and SCLC)). The table summarizes the evaluation metrics obtained for each model on the test set, highlighting their ability to differentiate between healthy cases and two major lung cancer subtypes.
